# Characteristics of Adolescents Affected by Mass Psychogenic Illness Outbreaks in Schools in Nepal: A Case-Control Study

**DOI:** 10.3389/fpsyt.2020.493094

**Published:** 2020-11-17

**Authors:** Ram P. Sapkota, Alain Brunet, Laurence J. Kirmayer

**Affiliations:** ^1^Research Centre of the Douglas Hospital, Department of Psychiatry, McGill University, Montreal, QC, Canada; ^2^Division of Social and Transcultural Psychiatry, Global Mental Health Program, McGill University, Montreal, QC, Canada; ^3^Culture and Mental Health Research Unit, Institute of Community & Family Psychiatry, Jewish Genera Hospital and Lady Davis Institute, Montreal, QC, Canada

**Keywords:** mass psychogenic illness, emotional contagion, childhood trauma, spirit possession, Nepal, dissociation, hypnotizability

## Abstract

This paper presents the first systematic case-control study of correlates of mass psychogenic illness (MPI) in an adolescent school population. MPI is generally construed as a dissociative phenomenon spread by social contagion to individuals who are prone to dissociation. We sought to test if the correlates of dissociative experiences most commonly proposed in the literature could predict caseness among students affected by episodes of mass psychogenic illness occurring in schools in Nepal. We assessed 194 cases and 190 controls (*N* = 384) of ages 11–18 years from 12 public schools. Cases and controls were comparable on all demographic variables, except for family configuration, with nuclear families more common among those affected. In bivariate comparisons, caseness was associated with childhood physical neglect and abuse, as well as living in nuclear families, peritraumatic dissociation, dissociative tendencies, and depressive and post-traumatic stress symptoms. Hypnotizability emerged as the strongest correlate of psychogenic illness among the cognitive and personality trait variables. However, in multivariable logistic regression, the correlates of dissociation did not predict caseness, suggesting that they do not adequately account for the phenomenon of mass psychogenic illness. An *ad-hoc* Classification and Regression Trees analysis showed that if an adolescent was highly hypnotizable and reported high rates of peritraumatic dissociative experiences, then there was a 73% probability of being a case in a mass psychogenic illness episode. Future studies involving other psychological, social and cultural factors, as well as school- and family-related factors are needed to understand the correlates of mass psychogenic illness and guide prevention and intervention.

## Introduction

In this paper, we present the first systematic case-control study of correlates of mass psychogenic illness (MPI) in an adolescent school population. MPI, also known by other names, including mass hysteria, mass sociogenic illness, mass conversion disorder, hysterical contagion, and medically unexplained epidemic illness, has been defined as the acute onset and rapid spread of constellations of symptoms suggestive of an organic/neurological illness but without an identifiable pathogen or medical cause, and which are therefore assumed to be of psychogenic origin (1, 2). The symptoms and mechanisms of spread of MPI may vary across social and cultural settings (3–6). For example, MPI outbreaks may involve different processes in a cohesive community or in an institutional setting (i.e., school, factory), where people are familiar with each other, have close ties, and share similar worldviews compared to outbreaks in settings where people are not familiar with each other and may not have similar worldviews (i.e., airports, bus/train stations) [see: (6, 7)]. A typical episode of MPI begins with an individual showing signs and symptoms of an illness such as headache, dizziness, nausea, abdominal pain, weakness, hyperventilation, fits, trance states, and fainting attacks or other symptoms that are reflective of the perceived threat or presumed cause of the illness (5, 8). In the beginning, the outbreak may be confined to a small and close group of people with “similar risk profiles” [(9), p. 912]. However, over time, the contagion spreads to affect a large number of people (10). This contagion effect is influenced by witnessing the behavior of afflicted individuals or by the communication of rumors and stories of the outbreak through word of mouth or popular media (1, 4, 6, 7, 11, 12).

MPI are thought to involve mechanisms of social contagion and dissociation. Dissociative phenomena affecting groups of people have been observed throughout the world for centuries (1, 7, 11). Written accounts of tarantism, dancing mania, and episodes of demonic possession in Europe date back to the Middle Ages [e.g., (13, 14)]. Both spirit possession and dancing mania have been interpreted as dissociative phenomena [e.g., (15–17)]. Although MPI outbreaks can involve a range of psychological symptoms, behaviors, and medically unexplained somatic symptoms [e.g., (18–20)], episodes involving dissociative trance and spirit possession states are common in many parts of the world [e.g., (21–23)].

Researchers have identified several characteristic features of MPI that are often applied to differentiate between MPI and other epidemics, including: lack of plausible pathophysiological explanation for the presenting symptoms; rapid spread and rapid remission of symptoms; occurrence mainly among young females; transmission of illness through visual and/or auditory exposure; and presence of stress associated with an actual or rumored catastrophic event (1, 2, 24). However, there are no pathognomonic diagnostic features because exceptions are found for all identified characteristics (25).

### Mass Psychogenic Illness in Nepal

MPI is a common occurrence in contemporary Nepal. Since the late 1990s, numerous schools and communities have been affected by outbreaks of unintentional dissociative trance^1^ (“marked alteration of consciousness or loss of the usual sense of identity without replacement by an alternate identity”) and/or possession states (“replacement of the usual sense of identity by that attributed to the possessing force”) [(26), p. 173]. In the last decade, more than 130 schools in at least 40 out of 77 districts in the country have witnessed epidemics of *chhopne* or *chhopuwā* (lit., “to catch, to get hold of, and to cover by someone or something”; spirit possession), affecting hundreds of pre-adolescent and adolescent children (27). These episodes typically involve multiple children in the classroom or school yard falling to the ground writhing, moaning, shouting and crying for periods of minutes to hours. Case studies conducted in Nepal indicate that individuals report significant distress, with both somatic and psychological symptoms, during and after the *chhopne* episode (20, 28, 29). The features of MPI episodes among young and adolescent children in schools in Nepal are consistent with the characteristics of MPI identified in the literature [e.g., (1)]: episodes typically begin with a single student affected by motor symptoms of conversion, dissociative trance and/or possession states, which spreads to other fellow students over a period of several days. MPI episodes may continue for a few weeks to months and may resolve on their own or after a traditional healing ritual, after intervention by psychosocial counselors (provided by non-profit organizations).

To date, four studies have examined the relationships between mental illness, trauma exposure, and MPI in Nepal. In a case-control study, Van Ommeren et al. (20) identified trauma, early loss and recent loss as predictors of MPI in a Bhutanese refugee camp located in Nepal. Shakya (28) found that dissociative trance behavior among girls exposed to a school epidemic was associated with low socioeconomic status, but did not find evidence that psychological distress had “triggered” the behavior in most cases. Sharma et al. (29) found associations between low academic performance, exposure to violence, mental illness (including anxiety and depression) and susceptibility to a fainting epidemic in a school in a village in Nepal. Finally, Sapkota et al. (23), in a mixed-methods case-control study of individuals in one affected village, found associations between epidemic spirit possession and both generalized anxiety and post-traumatic stress (PTSD); however, qualitative data suggested that possession was more likely an avenue to cope with and communicate distress associated with existing psychosocial problems than a reflection of mental illness.

Despite growing recognition of and interest in MPI in Nepal in the last decade, and efforts by the Ministry of Health and some non-profit organizations to prevent and treat MPI outbreaks, many schools still are affected each year, and some schools have been recurrently affected by MPI episodes for 10–12 years. Effective prevention and management of MPI requires better understanding of its causes and correlates.

In an earlier study in an adolescent school population in Nepal, Sapkota et al. (30) examined if factors associated with the two most widely used etiological models of dissociation (i.e., trauma and socio-cognitive models) [e.g., see (31–36)] and other measures of distress (i.e., current psychological distress, quality of life, PTSD symptoms, and depressive symptoms) could predict the level of dissociative experiences (30). The current study extended this research by examining an adolescent population affected by MPI.

The present study aimed to advance understanding of the potential causes and correlates of MPI as a means to better predict its course and inform intervention guidelines and prevention strategies. We hypothesized that the factors implicated in the trauma and the socio-cognitive models and other correlates of dissociative experience and behaviors commonly proposed in the dissociation literature would predict caseness in MPI episodes occurring among adolescents in schools in Nepal. To test this, we compared MPI-affected (i.e., cases) and controls on measures of four sets of potential explanatory factors: (a) childhood trauma (i.e., physical abuse, sexual abuse, emotional abuse, physical neglect, emotional neglect); (b) cognitive and personality traits (i.e., hypnotizability, fantasy proneness, susceptibility to emotional contagion, susceptibility to cognitive failures); (c) current distress (i.e., quality of life, depressive symptoms, PTSD symptoms); and (d) trait and state dissociative experiences and behaviors (i.e., dissociative tendency and peritraumatic dissociation).

### Why Consider Factors Associated With Dissociation as Potential Predictors of Caseness in MPI?

The DSM-5 defines dissociation as “a disruption of and/or discontinuity in the normal integration of consciousness, memory, identity, emotion, perception, body representation, motor control, and behavior” [(37), p. 291]. Dissociative trance and/or possession states are among the main presenting features of MPI in schools in Nepal. Frequent trance-like behaviors have been identified as “the single best predictor of a dissociative disorder” in children [(38), p. 42]. Trance and possession episodes that occur outside ritual contexts, that are unintended, and are associated with distress and impairment in daily functioning are recognized as dissociative disorders by ICD-11 (39) and the DSM-5 also includes spirit possession as a cultural variant of dissociative identity disorder (37). Children affected by MPI in schools in Nepal also have various characteristics that closely resemble the elements of current ICD and DSM diagnoses of dissociative disorders. First, they experience disruptions in perception. Some affected children report that immediately before collapsing to the ground in a trance-like state, they see someone (usually an unidentified black figure, or a “ghost-like” figure) trying to strangle them or take them away by pulling them by the hand. Others see the spirits of deceased women who died by suicide or other unnatural causes (usually presenting as a white figure). Second, the children experience disintegrated memory. They usually do not remember what they did or what happened during the episode of dissociative trance or spirit possession. Third, they experience alterations in identity and/or awareness. Affected children are either possessed by spirits and behave accordingly (e.g., if they are possessed by *nāg devatā* [the snake god], they crawl like a snake on the ground) or collapse in a trance-like state and do things that they would not do in a “normal” conscious state, such as scolding, hitting or spitting at teachers and friends. Finally, affected children display disruptions in behavior—for example, screaming, continuously weeping, running around for no apparent reason, and so forth—which they disavow after returning to awareness (27).

It is important to note, however, that in making a comparison with conversion and dissociative disorders and referencing ICD and DSM, we do not suggest that *chhopne* episodes in Nepal are instances of individual psychopathology [see (40–43)]. Rather, we draw this comparison in order to suggest that *chhopne* may involve similar, though culturally patterned, correlates and underlying processes and mechanisms to those of dissociative phenomena (44). Individuals with pre-existing social stress, emotional distress, or psychopathology may be at greater risk to respond to the spread of MPI with similar symptoms.

## Materials and Methods

### Participants and Procedure

This is a case-control study of adolescents who had or had not experienced *chhopne* during a recent MPI outbreak at their school. Data were collected from 12 public schools in the rural areas of Sindhuli, Sindhupalchowk, Dang, Dolkha, and Ramechhap districts from August to October 2015. With an exception of Dang district, MPI episodes were actively occurring during the time of this study. In Dang district, MPI episodes had stopped 6 to 12 months prior to the assessment. MPI affected schools were identified via the national newspapers, and by word of mouth. The final sample comprised 194 cases and 190 controls (*N* = 384).

The schools' administration teams prepared lists of students affected by MPI. All participants and their parents or caregivers were involved in the informed consent process. Considering low literacy of the parents/caregivers in rural settings of Nepal, we obtained verbal/oral consent of the parents or caregivers and with the agreement of the parents/caregivers, both the principal of the school, as a common guardian of the children during school hours, and the class teacher or an administrative staff, as a witness, provided written consent on the day of the interview. Students themselves provided informed assent. This is a common practice for research in Nepal (45).

The following inclusion/exclusion criteria were used to select the cases: (1) 11–18 years of age; (2) recently experienced at least one episode of *chhopne* (as defined by teachers, children themselves, family members and/or traditional healers); and (3) not suffering from epilepsy or other severe ailments (as defined by self-report). All the affected students present in the school on the day of the interview who met inclusion criteria were invited to participate in the study.

Following the strategy adopted by Van Ommeren et al. (20), the control group was formed by asking participants to identify a friend in their class who had not suffered from *chhopne* during the epidemic or in the past. The control group consisted mostly of close friends of the affected children. They had comparable demographics and similar exposure to *chhopne* episodes but had never experienced *chhopne* themselves. In situations where the close friend of the affected child was absent from class on the day of the interview, the affected child and/or the schoolteacher identified another participant for the control group.

The Research Ethics Committee of the Jewish General Hospital, Montreal Canada, provided ethical approval for this study. No financial support was provided to the participants. Snacks were provided to each participant after administering the questionnaire.

### Measures

Three of the study instruments (the Brief Childhood Trauma Questionnaire, the Depression Self-Rating Scale and the Child PTSD Symptom Scale) were previously translated to Nepali by others and validated for use in Nepal (45, 46). All other questionnaires used in this study were translated and adapted in the previous study (30) using mixed methods to achieve semantic, content, technical, and criterion equivalence (47). Briefly, two Nepali Social Work graduates who work as professional translators in Nepal performed the translation of the instruments. These translators had extensive experience in translating terminology related to psychosocial and mental health and psychometric measures from English to Nepali and vice versa. In the second step, two experienced Nepali psychosocial counselors and a psychologist together reviewed the translated instruments. The counselors were instructed to appraise the instruments on the comprehensibility of the language used, acceptability of the items and the response set (i.e., yes/no, Likert-type) for each item, as well as the meaning and relevance of the questions in the local culture. Following the comments and recommendations of the counselors and the psychologist, the instruments were revised by the investigators. Third, a focus group discussion about the instruments was conducted in Dang district with four children (two boys and two girls) aged 12–15 years. The instruments were further modified based on the suggestions of the children. In the fourth step, the revised Nepali instruments were translated back into English by two translators blind to the original English version of the instruments. In the fifth step, a native English-speaking graduate student with good knowledge of spoken Nepali and past experience with ethnographic research on mental health in Nepal compared the back-translations with the original English items to identify any errors in translation or misunderstanding of English idioms by the Nepali translators. In the sixth step, to evaluate the completeness of the translation, the issues identified by the native English speaker, the mental health workers, and the children in the focus group discussion were reviewed by a team comprising two translators, a native English speaker, and a Nepali psychologist. Some issues raised in the previous steps could not be resolved through discussion among the team (i.e., What to do with the items identified as irrelevant for the rural contexts? What to do with items related to sexual abuse [e.g., Someone tried to make me do/watch sexual things]?). To address these issues, four additional focus group discussions with 16 children (8 boys and 8 girls) aged 12–17 years were conducted in a residential school setting in Bhaktapur district. Based on the information from these focus groups instruments were revised and finalized. The translation and adaptation process was completed over a period of 1 year (from July 2014 to August 2015). All the measures except for the Depression Self-Rating Scale had good psychometric properties (Cronbach's alpha = 0.74–0.94; intra-class correlation coefficient = 0.72–0.89) in the previous as well as the current study [see (30)]. Cronbach's alpha for each measure in this study is reported.

*The Adolescent Dissociative Experience Scale (A-DES)* (48) is a 30-item Likert-type screening measure assessing dissociative experiences among adolescents. Research participants rate each statement on a scale of 0 (never) to 10 (always). A mean score >3.7 indicates significant dissociation (48). For the A-DES, the Cronbach's alpha (49) was 0.92.

The *Creative Experiences Questionnaire* (CEQ) (50) is a 25-item yes/no self-report questionnaire measures the tendency to have frequent and intense involvement in fantasy and daydreaming. For the CEQ, the Cronbach's alpha was 0.75.

The *Emotional Contagion Scale* (ECS) (51) is a 15-item measure of susceptibility to the influence of “other's emotions.” Participants rate their response to each item on a 4-point scale ranging from 1 (Never) to 4 (Always). The total score is computed as a sum of all items, and ranges between 15 and 60. For the ECS, the Cronbach's alpha was 0.76 in this study.

The *Cognitive Failures Questionnaire* (CFQ) (52) is a 25-item self-report inventory that assesses an individual's tendency to failures in ordinary memory, perception, and motor function in everyday life (52). Respondents rate each item on a 5-point Likert-type scale of frequency over the last 6 months. The total score is obtained by summing responses on all the items. For the CFQ, the Cronbach's alpha was 0.84.

The *Comprehensive Quality of Life–School Version* (ComQol-S5) (53) is a 35-item measure of quality of life for children and adolescents 11–18 years of age. In this study, we only used the 21 items of the objective dimension of quality of life (for seven domains). For the ComQol-S5, the Cronbach's alpha was 0.77.

The *Depression Self-Rating Scale* (DSRS) (54) is an 18-item self-report measure of depressive symptoms from the past week designed for children and adolescents. Responses include: 0 (mostly), 1 (sometimes), and 2 (never). Total score is computed as a sum of responses of all the items after reverse coding the negative items. For the DSRS, the Cronbach's alpha was 0.53. Internal consistency statistics in the current study, as well as in our previous study conducted in Nepal (30), have consistently found poor psychometrics for DSRS. Therefore, results involving DSRS should be interpreted cautiously.

The *Brief Childhood Trauma Questionnaire* (BCTQ) (55) has 28-items that allow for retrospective identification of child abuse and neglect among adolescents and adults. Respondents rate each item using a 5-point scale with response options ranging from 1 (never true) to 5 (very often true). Only 25-items pertaining to five abuse and neglect subscales were used in the analysis. For the BCTQ, the Cronbach's alpha was 0.89.

The *Peritraumatic Dissociative Experiences Questionnaire* (PDEQ) (56) is a 10-item self-report measure that retrospectively evaluates the extent of dissociation at the time of a traumatic event. Each item is scored from 1 (not at all true) to 5 (extremely true). A summed score above 15 indicates clinically significant dissociation. Participants were asked to think of a traumatic event (“*dukhad/man ma gahiro chot parne ghatana”* in Nepali) and to respond to each item based on the experiences they had during and/or immediately after this event. The same event was used to assess traumatic stress (i.e., PTSD) symptoms. For the PDEQ, the Cronbach's alpha was 0.81.

The *Child PTSD Symptom Scale* (CPSS) (57) was developed as a child-version of the Post-traumatic Diagnostic Scale (57). The CPSS has two parts: The first part contains 17 items that correspond to the PTSD diagnostic criteria in DSM-IV; the second part includes 6 items related to impairment in functioning. Items are scored on a scale of 0 (not at all) to 4 (almost always) based on frequency of experience over the past week. Only the first part (17-items) of the scale was used in the present study (alpha of 0.87).

The *Harvard Group Scale of Hypnotic Susceptibility* (HGSHS), Form A (58) consists of 11 sets of instructions/suggestions aimed at inducing hypnotic trance. After going through the 11 suggestions, participants judge whether or not they performed the suggested behavior in a response booklet. The amnesia item (the 12th item on the scale) is scored based on the participant's recall of the nine activities suggested during hypnosis. Amnesia is scored positive “if fewer than four of the nine items induced within hypnosis were recalled before the signal to remember was given” [(58), p. 12]. The hypnotic induction procedures were voice recorded from a Nepali version of HGSHS that was translated and adapted as a part of this study. The recording of the hypnotic induction procedures was played after a brief introduction by the researcher in all of the hypnotic induction sessions conducted with the research participants. The introduction involved telling the participants what the session was about and how it would proceed. Hypnosis (*sammohan* in Nepali) and the hypnotic induction process were introduced as an attention focusing exercise (*dhyān kendrit garne abvyās*) because the participants were unfamiliar with the concept of hypnosis. Thus, to elicit maximum engagement with the task, we introduced a term that the participants were familiar with and would be more engaging and non-threating. Also, an unfamiliar female voice was used for the recording of instructions for the induction to make it less authoritative and more relaxing. For the HGSHS, the Cronbach's alpha was 0.65.

### Data Analysis

Data were analyzed using IBM SPSS Statistics 23.0 (IBM, Armonk, N.Y.). Bivariate correlation between the independent variables and a series simple binary logistic regression analysis (i.e., involving one independent variable and one binary dependent variable) for each variable of interest were conducted. Four separate multivariable binary logistic regression analysis were conducted by grouping the variables as childhood trauma, cognitive and personality traits, current level of distress, or a specific propensity for dissociative experience and behaviors. In the final model, all the variables were simultaneously included. Model fit was assessed using the Hosmer and Lemeshow goodness-of-fit χ^2^ statistic. A non-significant (i.e., *p* > 0.05) Hosmer and Lemeshow χ^2^ statistic indicates a good fit (59). A Classification and Regression Trees (CART) analysis was conducted to explore the associations between independent variables and caseness taking into account subgroup characteristics and potential interactions among the independent variables.

## Results

### Preliminary Analysis and Socio-Demographic Characteristics

The final dataset included 193 MPI affected and 186 not affected adolescents (*N* = 379). Of the 384 study participants, 5 individuals were excluded: one individual had incomplete data because of a *chhopne* episode during the interview and four others were aged <11 years old. Less than 1% (0.75%) of the data were missing. Based on the MCAR test (χ^2^ = 276.73, *df* = 254, *p* = 0.156), missing data were determined to be missing at random, and therefore were imputed using the EM algorithm (60, 61).

Table 1 displays the socio-demographic characteristics of the case and control groups. There were no statistically significant differences between the groups in age, gender, caste/ethnicity, socio-economic status, or level of education, except for family type (χ^2^ = 3.9, *df* = 1, *p* = 0.04). A majority (55.5 %) of the affected were living in nuclear families, while a majority (54.8%) of the controls were living in united/extended families. Children living in a nuclear family had 1.5 times higher odds of being affected by MPI (see **Table 3**). Demographic variables including sex, age, ethnicity, language, and level of education, marital status, residence, and family economic status did not independently discriminate between the cases and the controls and had trivial effect sizes (*d* < 0.10) according to Cohen's (62) guidelines. The vast majority of study participants were female (96.4%), with a male/female ratio of about 1:31 among those affected.

**Table 1:** Demographic characteristics of respondents in case and control groups.

**Variables**	**Subcategory**	**Control**		**Case**		**Total**	***χ2***	***df***	***p***
		***n***	**%**	***n***	**%**				
Age group	Early adolescents	124	48.1	134	51.9	258	0.33	1	0.56
	Adolescents	62	51.2	59	48.8	121			
Gender	Male	7	53.8	6	46.2	13	0.12	1	0.47
	Female	179	48.9	187	51.1	366			
District	Sindhuli	64	50	64	50	128	0.59	4	0.96
	Sindhupalchowk	24	51.1	23	48.9	47			
	Dang	31	50	31	50	62			
	Ramechhap	49	48.5	52	51.5	101			
	Dolkha	18	43.9	23	56.1	41			
Education	Lower secondary	111	51.6	104	48.4	215	1.29	1	0.25
	Secondary	75	45.7	89	54.3	164			
Ethnicity	Brahman/Chhetri	74	48.7	78	51.3	152	2.09	2	0.35
	Janjati	82	52.6	74	47.4	156			
	Dalit	30	42.3	41	57.7	71			
Religion	Hindu	161	49.4	165	50.6	326	0.84	2	0.66
	Buddhist	18	51.4	17	48.6	35			
	Others	7	38.9	11	61.1	18			
Family occupation	Agriculture	139	46.8	158	53.2	297	3.33	4	0.51^*^
	Service (Job)	7	53.8	6	46.2	13			
	Business	18	54.5	15	45.5	33			
	Working abroad	18	62.1	11	37.9	29			
	Others	4	57.1	3	42.9	7			
Family type	Nuclear	94	44.5	117	55.5	211	3.9	1	0.04
	Joint/Extended	92	54.8	76	45.2	168			
Residence	Rented house	2	20	8	80	10	3.47	1	0.11^*^
	Family owned	184	49.9	185	50.1	369			
Perceived SES	Low	61	49.6	62	50.4	123	0.02	1	0.89
	Medium	125	48.8	131	51.2	256			

* *Fisher's exact test or fisher-freeman-halton exact test*.

### Bivariate Analysis

Table 2 displays the bivariate Pearson correlation matrix for all main variables and five subscales of childhood trauma. Socio-demographic variables (i.e., age, gender, caste/ethnicity, mother tongue, level of education, marital status) were not included in the matrix because these variables were not significantly associated with case status. Results indicated significant positive associations between all variables, except between Quality of Life and Emotional Neglect, and dissociative experiences and behaviors as measured by the A-DES. Following 61 guidelines for effect size, post-traumatic stress [r = 0.33], fantasy proneness [r = 0.35], and cognitive failures [r = 0.39] each had medium effects on dissociative experience, while all other variables had small effect size. However, results of the point biserial correlation (r_pb_) (not included in Table 2) show that only a few variables have small but significant association with case status (e.g., dissociative experience [r_pb_ = 0.17], peritraumatic dissociation [r_pb_ = 0.19], post-traumatic stress [r_pb_ = 0.14], depressive symptoms [r_pb_ = 0.12], childhood trauma [r_pb_ = 0.10], hypnotizability [r_pb_ = 0.29]). The data indicate that majority of the variables included in the correlation analysis are correlates of dissociative experience (i.e., A-DES) and case status in MPI episodes, but the strength of the relationship is weak.

**Table 2:** Pearson product-moment correlation coefficients between main variables and five subscales of childhood trauma.

**Variables**	**A-DES**	**PD**	**QoL**	**PTS**	**DS**	**FP**	**CF**	**EC**	**PN**	**EN**	**EA**	**SA**	**PA**	**CT**
Dissociation (A-DES)	–													
Peritraumatic Dissociation (PD)	0.26^**^	–												
Quality of Life (QoL)	0.04	0.03	–											
Post-traumatic Stress (PTS)	0.33^**^	0.47^**^	−0.06	–										
Depressive Symptoms (DS)	0.19^**^	0.23^**^	−0.35^**^	0.41^**^	–									
Fantasy Proneness (FP)	0.35^**^	0.24^**^	−0.06	0.29^**^	0.24^**^	–								
Cognitive Failures (CF)	0.39^**^	0.30^**^	−0.13^*^	0.45^**^	0.34^**^	0.35^**^	–							
Emotional Contagion (EC)	0.23^**^	0.34^**^	0.01	0.39^**^	0.28^**^	0.36^**^	0.43^**^	–						
Physical Neglect (PN)	0.18^**^	0.16^**^	−0.29^**^	0.28^**^	0.37^**^	0.22^**^	0.31^**^	0.18^**^	–					
Emotional Neglect (EN)	0.06	0.04	−0.34^**^	0.10	0.31^**^	0.03	0.17^**^	0.02	0.48^**^	–				
Emotional Abuse (EA)	0.26^**^	0.32^**^	−0.25^**^	0.54^**^	0.32^**^	0.15^**^	0.43^**^	0.21^**^	0.50^**^	0.35^**^	–			
Sexual Abuse (SA)	0.19^**^	0.26^**^	−0.10^*^	0.26^**^	0.13^*^	−0.01	0.24^**^	0.19^*^	0.31^**^	0.19^**^	0.59^**^	–		
Physical Abuse (PA)	0.15^**^	0.27^**^	−0.17^**^	0.46^**^	0.23^**^	0.14^**^	0.25^**^	0.19^**^	0.45^**^	0.25^**^	0.72^**^	0.61^**^	–	
Childhood Trauma (CT)	0.23^**^	0.29^**^	−0.31^**^	0.44^**^	0.36^**^	0.14^**^	0.38^**^	0.20^**^	0.72^**^	0.60^**^	0.85^**^	0.73^**^	0.82^**^	–
Hypnotizability	0.15^*^	0.10	0.13	0.25^**^	0.02	0.06	0.07	0.19^**^	−0.05	−0.17^*^	−0.03	−0.01	−0.01	−0.08

* *p < 0.05*;

** *p < 0.01*.

### Regression Analysis

Potential predictors of caseness in MPI outbreaks were examined in a series of simple logistic regressions (Table 3). Then, the variables were grouped into four separate models based on theoretical approaches to MPI in the literature, and tested in multivariable logistic regression analysis as shown in Table 4.

**Table 3:** Simple binary logistic regression analysis of predictors of MPI caseness^*^.

**Variables**	**B (S.E.)**	**Wald *χ^2^***	***p***	**OR**	**95% CI**	
					**Lower**	**Upper**
Family type (0 = Nuclear, 1 = Extended)	0.41 (0.21)	3.89	0.05	1.51	1.01	2.26
Physical neglect	0.06 (0.03)	3.89	0.05	1.06	1.00	1.12
Emotional neglect	0.01 (0.03)	0.02	0.89	1.01	0.95	1.06
Emotional abuse	0.02 (0.03)	0.88	0.34	1.02	0.97	1.08
Sexual abuse	0.04 (0.03)	2.82	0.09	1.04	0.99	1.10
Physical abuse	0.07 (0.03)	6.97	0.01	1.07	1.02	1.13
Childhood trauma	0.01 (0.01)	3.90	0.05	1.01	1.00	1.03
Fantasy proneness	0.03 (0.02)	2.23	0.13	1.03	0.99	1.08
Cognitive failures	0.01 (0.01)	0.84	0.36	1.01	0.99	1.02
Emotional contagion	0.02 (0.01)	1.31	0.25	1.02	0.99	1.04
Depressive symptoms	0.07 (0.03)	5.43	0.02	1.07	1.01	1.13
Peritraumatic dissociation	0.05 (0.02)	9.51	0.01	1.05	1.02	1.09
Post-traumatic stress	0.03 (0.01)	4.99	0.03	1.03	1.01	1.06
Hypnotisability	0.19 (0.06)	9.73	0.01	1.21	1.07	1.36
Quality of life	0.01 (0.02)	0.31	0.58	1.01	0.97	1.05
Dissociation	0.13 (0.06)	5.01	0.02	1.13	1.02	1.27

* *Dependent variable: Control = 0, Cases = 1*.

**Table 4:** Multiple logistic regression analysis for childhood trauma (Model 1), current distress (Model 2), cognitive and personality traits (Model 3) and dissociative experiences and behaviors (Model 4) of case-control groups.

	**Variables**	**B (S.E.)**	**Wald *χ^2^***	***p***	**OR**	**95% CI**	
						**Lower**	**Upper**
Model 1	Emotional abuse	−0.07 (0.04)	2.82	0.09	0.93	0.86	1.01
	Sexual abuse	0.02 (0.03)	0.32	0.57	1.02	0.95	1.09
	Physical abuse	0.09 (0.04)	5.12	0.02	1.10	1.01	1.19
	Physical neglect	0.06 (0.04)	2.40	0.12	1.06	0.99	1.14
	Emotional neglect	−0.03 (0.03)	0.63	0.43	0.98	0.92	1.04
Model 2	Depression	0.04 (0.04)	0.98	0.32	1.04	0.96	1.12
	Post-traumatic stress	0.03 (0.02)	2.77	0.10	1.03	1.00	1.06
	Quality of life	0.03 (0.03)	0.91	0.34	1.03	0.97	1.08
Model 3	Hypnotizability	0.19 (0.06)	9.18	0.01	1.21	1.07	1.37
	Fantasy proneness	−0.02 (0.04)	0.34	0.56	0.98	0.91	1.06
	Cognitive failures	−0.02 (0.01)	2.16	0.14	0.98	0.96	1.01
	Emotional contagion	0.03 (0.02)	1.18	0.28	1.03	0.98	1.07
Model 4	Dissociation	0.06 (0.07)	0.87	0.35	1.06	0.93	1.21
	Peritraumatic dissociation	0.05 (0.02)	7.58	0.01	1.05	1.01	1.09

### Model 1: Childhood Trauma and Abuse as Predictors of MPI Case Status

Childhood trauma (as a composite variable) was significantly associated with MPI case status, χ^2^ (1) = 3.97, *p* < 0.05, in a binary logistic regression analysis. A multivariable logistic regression analysis with five variables (physical neglect, emotional neglect, emotional abuse, physical abuse, and sexual abuse) showed that only physical abuse significantly predicted case status, Wald's χ^2^ (1, *N* = 379) = 5.12, *p* < 0.05 (see Table 4). The overall model was significant, χ^2^ (5) = 11.99, *p* = 0.035, with a Hosmer and Lemeshow goodness-of-fit test of, χ^2^ (8) = 9.10, *p* = 0.334. Excluding physical abuse resulted in a non-significant overall model, χ^2^ (4) = 6.76, *p* = 0.149, confirming that physical abuse was the only significant predictor of caseness in MPI.

### Model 2: Current Distress and Case Status in MPI

In the simple binary logistic regression, depressive symptoms (Wald's χ^2^ (1, *N* = 379) = 5.43, *p* < 0.05, and post-traumatic stress, Wald's χ^2^ (1, *N* = 274) = 4.99, *p* < 0.05), were significant predictors of caseness while the quality of life was not, Wald's χ^2^ (1, *N* = 379) = 0.311, *p* = 0.58. A multivariable logistic regression performed with all three variables as predictors, the overall model was non-significant, χ^2^ (3) = 6.52, *p* = 0.089, and none of its predictors were statistically significant at *p* < 0.05 (see Table 4).

### Model 3: Cognitive and Personality Traits and Case Status in MPI

A multivariable logistic regression conducted with hypnotizability, fantasy proneness, cognitive failures, and emotional contagion as the predictors and MPI case status as the outcome was statistically significant. However, only hypnotizability, Wald's χ^2^ (1, *N* = 195) = 9.18, *p* < 0.05, was statistically significant. Omitting hypnotizability resulted in a non-significant overall model, χ^2^ (3, *N* = 379) = 2.71, *p* = 0.438. These variables were not significant even in a simple binary logistic regression analysis. This result suggests that commonly used measures of fantasy proneness, cognitive failures and emotional contagion are unrelated to MPI caseness.

### Model 4: Dissociative Experience and MPI Caseness

Assessed individually in simple logistic regression models, both trait dissociation, Wald's χ^2^ (1) = 5.01, *p* < 0.05, and peritraumatic dissociation, Wald's χ^2^ (1) = 9.51, *p* < 0.05, were statistically significant predictors of case status. However, in a model involving both variables, only peritraumatic dissociation remained statistically significant (see Table 4), suggesting that trait dissociation is not a robust predictor of MPI caseness.

### Final Model: Classification and Regression Trees (CART) Analysis

**Figure 1:**
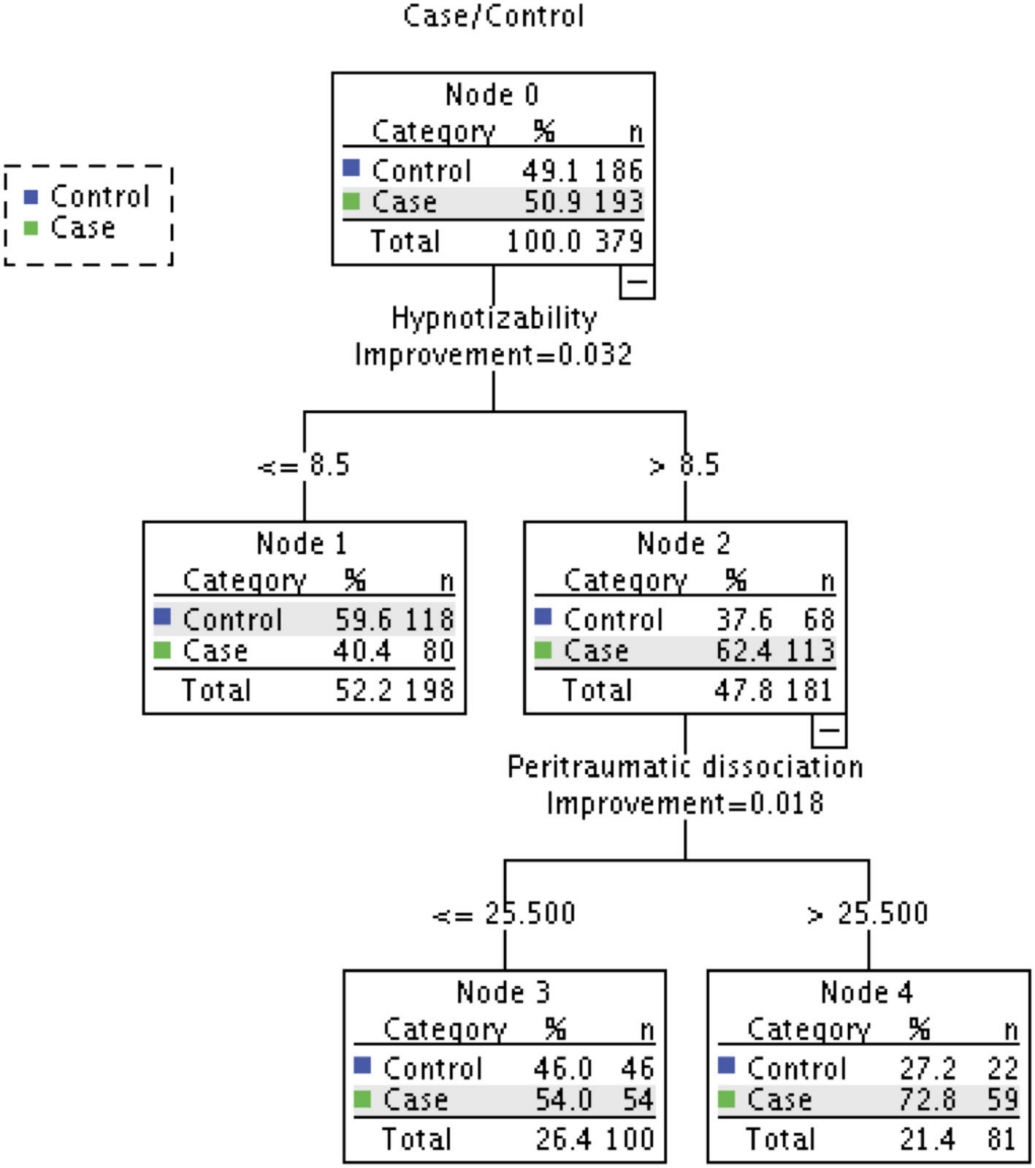
Classification tree produced by CART analysis. Dependent variable: Case/Control; Independent variables: Physical Neglect, Emotional Neglect, Emotional Abuse, Sexual Abuse, Physical Abuse, Fantasy Proneness, Cognitive Failures, Emotional Contagion, Depression, Peritraumatic Dissociation, Post-traumatic Stress, Hypnotizability, Quality of Life, Dissociative experience. Node 0 contains frequency counts and percentages of all observations in the model, while all other nodes display the number and percentage of participants in that particular subgroup and the percentage of accurate classification of group membership (i.e., case or control) by that particular subgroup of a variable.

When all the variables in the four models were simultaneously entered into the final multiple logistic regression model, no variable showed a significant effect on predicting caseness (Table 5). We conducted CART analysis (63) to explore if any hidden associations based on subgroup characteristics or the interactions between independent variables substantially influenced the effect of other variables in predicting caseness. We included all the variables in the final model as predictor variables and case/control status as a dependent variable. Considering the relatively small sample size in this study for CART analysis, the minimum number of cases required for a split in the parent node and child node was set to 50 and 25, respectively. CART analysis performed using Gini criterion as a measure of node impurity produced a classification tree with 5 nodes and 3 terminal nodes. As seen in Figure 1, the CART analysis suggested that hypnotizability and peritraumatic dissociation were the most important predictors of caseness. This result is consistent with the results of logistic regression models (see Table 5).

**Table 5:** Multivariable logistic regression analysis with all variables (Control = 0, Cases = 1).

**Variables**	**B (S.E.)**	**Wald *χ^2^***	***p***	**OR**	**95% CI**	
					**Lower**	**Upper**
Family type (0 =Nuclear, 1 =Extended)	0.08 (0.44)	0.04	0.85	1.09	0.46	2.60
Physical neglect	−0.14 (0.09)	2.33	0.13	0.87	0.72	1.04
Emotional neglect	−0.01 (0.07)	0.01	0.91	0.99	0.86	1.14
Emotional abuse	−0.04(0.10)	0.19	0.66	0.96	0.79	1.16
Sexual abuse	−0.01 (0.08)	0.01	0.98	1.00	0.85	1.16
Physical abuse	0.13 (0.09)	2.37	0.12	1.14	0.96	1.36
Fantasy proneness	−0.07 (0.06)	1.27	0.26	0.93	0.82	1.05
Cognitive failures	−0.02 (0.02)	0.95	0.33	0.98	0.94	1.02
Emotional contagion	0.05 (0.04)	1.89	0.17	1.05	0.98	1.13
Depressive symptoms	0.07 (0.07)	1.00	0.32	1.07	0.93	1.23
Peritraumatic dissociation	0.08 (0.04)	3.52	0.06	1.08	1.00	1.18
Post-traumatic stress	−0.03 (0.04)	0.74	0.39	0.97	0.90	1.04
Hypnotizability	0.10 (0.09)	1.26	0.26	1.11	0.93	1.32
Quality of life	−0.03 (0.05)	0.51	0.47	0.97	0.88	1.06
Dissociation	−0.11 (0.13)	0.68	0.41	0.90	0.70	1.16
Constant	−0.18 (3.36)	0.01	0.96	0.83		
	***χ***^**2**^	***df***	***p***			
Overall Model	22.465	15	0.10			
Hosmer and Lemeshow	3.968	8	0.86			
Cox and Snell R^2^	0.173					
Nagelkerke R^2^	0.231					

*χ^2^, Chi square; df, Degrees of Freedom; OR, Odds Ratio; CI, Confidence Interval*.

**Figure 2:**
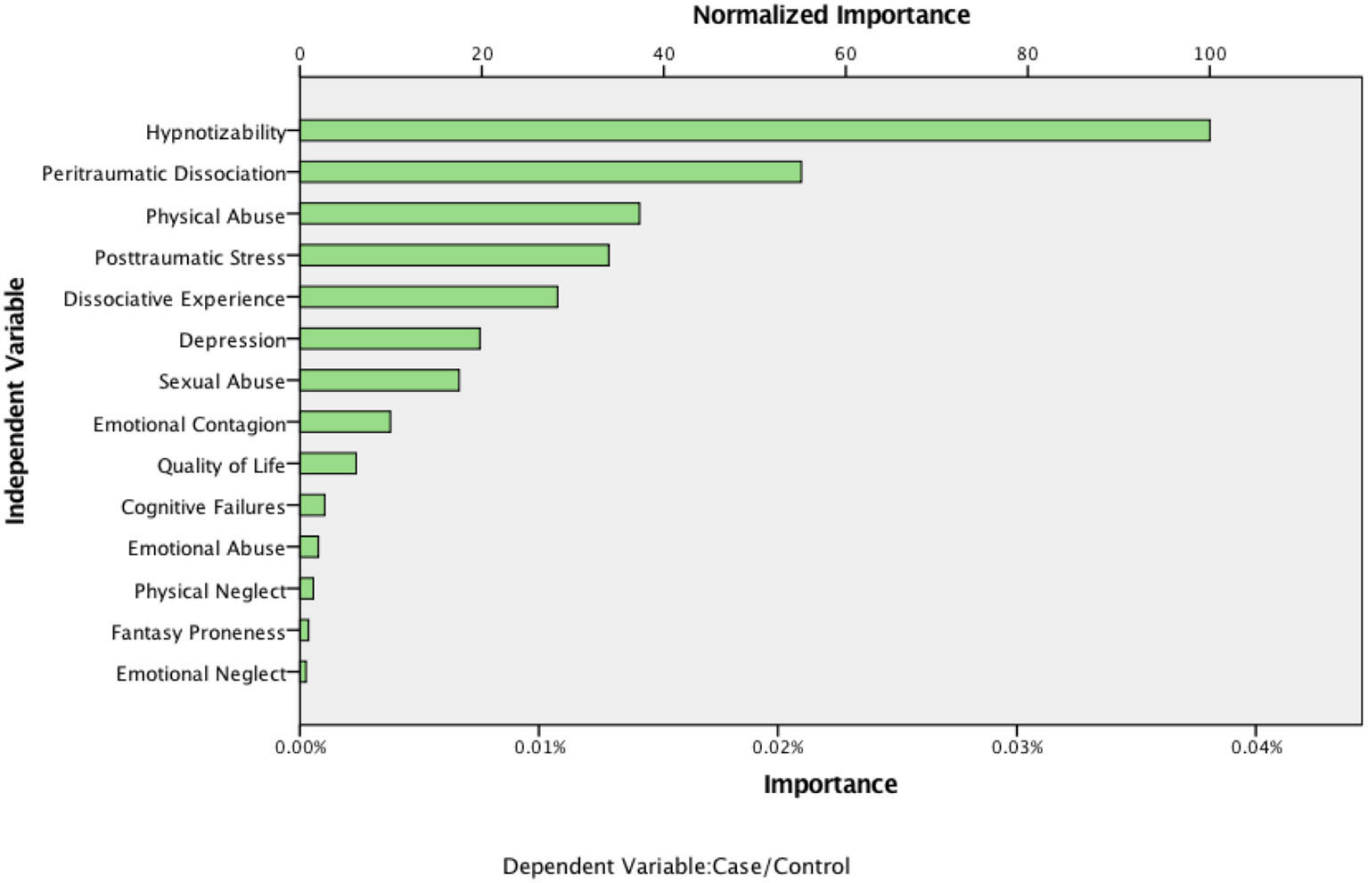
Importance of variables in the classification tree model in predicting case and control groups.

The overall classification accuracy achieved by the CART tree was 61%, which is lower than that obtained with multiple logistic regression (i.e., 67%). However, a useful finding from CART analysis based on subgroup interactions was that if an adolescent was highly hypnotizable (i.e., above 8.5 on the HGSHS) and reported a high rate of peritraumatic dissociation (i.e., >25 on the PDEQ), there was a is 73% probability they had experienced *chhopne* (see Node 2 and Node 4 in Figure 1). Further, CART analysis revealed that some predictor variables (i.e., sexual abuse, susceptibility to emotional contagion, quality of life) that were not significant even in bivariate comparisons, did play an important role in predicting *chhopne*. Consistent with the logistic regression findings, some variables (e.g., emotional neglect, fantasy proneness) remained least important (see Figure 2).

## Discussion

Using a case-control design, the present study tested the ability of multiple variables associated dissociative experiences and behaviors to predict caseness in MPI outbreaks among adolescents in Nepal. In a series of simple binary logistic regressions, the odds of being affected by MPI episodes were higher among those who were living in a nuclear family, reported physical abuse, physical neglect, depressive symptoms, post-traumatic stress symptoms, proneness for dissociative experiences, prior experience of peritraumatic dissociation, and higher hypnotic susceptibility, than among those without such experiences. However, although these variables were statistically significant predictors of caseness, the effect sizes were small (i.e., *d* = 0.1–0.2) for nuclear family and hypnotic susceptibility, and trivial (i.e., Cohen's *d* < 0.10) for physical abuse, depressive symptoms, post-traumatic stress, dissociative experience, peritraumatic dissociation. When these variables were entered in multiple regression models, only physical abuse, peritraumatic dissociation, and hypnotizability made unique contributions to differentiating MPI-affected from non-affected individuals.

In terms of correlations, the current study replicates the findings of our previous study with a different sample of “healthy” adolescents from the school population (30). In that study, A-DES was associated with childhood abuse, post-traumatic stress symptoms, cognitive failures, fantasy proneness. However, in the present study, these variables had either small or no effect on MPI case status. This challenges the assumption that the trance and possession episodes in MPI (which might be classified as dissociative disorders in the DSM and ICD) can be adequately explained by the same variables associated with dissociative symptoms and behaviors in the general adolescent population (as measured by the A-DES), that is cognitive failures, fantasy proneness, and post-traumatic stress (30). The A-DES itself had only a very weak relationship to case status in the current study further suggesting that the phenomenon of MPI has different determinants.

### Childhood Trauma

In this study, the correlation between childhood trauma and the measure of trait dissociation (A-DES) was not as strong as has been found in other studies [e.g., (64)]. This is consistent with our previous study of correlates of dissociation in a general adolescent population in Nepal (30). However, the present study did find that childhood trauma, especially physical abuse, was a robust predictor of caseness in MPI outbreaks in Nepal. Although studies assessing the effect of childhood abuse on case status in MPI are sparse, some studies have identified childhood trauma (i.e., death of a family member or close friends) and parental divorce, as significant predictors of MPI (20, 65). However, other studies have not found a significant association between MPI and traumatic events (66) or grief (67). Some studies have found physical abuse to be the strongest predictor of dissociative experiences and behaviors in children [e.g., (68–70)]. The finding that physical abuse is a predictor of MPI may shed some light on the high rates of MPI in Nepal. Corporal punishment as a means of disciplining children is common practice in Nepal. A recent survey found that 50% of children are physically punished in Nepal (71). It is very likely that what our study participants reported as childhood trauma (i.e., physical abuse) was for many an ongoing experience. It is therefore possible that MPI is response or coping mechanism associated with this abuse.

Living in a nuclear family was associated with MPI. It was not possible to explore family and school dynamics with the existing quantitative data. However, the transition from joint/extended families to nuclear families, which is occurring in many regions in Nepal due to labor migration and urbanization, may entail a loss of resources and social support. The lack of social and emotional support in childhood has been found to be associated with dissociative experiences and behaviors [e.g., (72, 73)]. A study of psychosocial problems (i.e., cognitive, emotional, and behavioral) among a randomly selected sample of adolescents from various schools of one district in Nepal found that children living in nuclear families with a single parent were about 3.5 times more likely to have psychosocial problems than those living in a non-nuclear family with both parents (74). Likewise, Kandel et al. (71) found that the odds of being physically punished were higher among children whose father was currently away from home (either abroad or elsewhere in Nepal). In a previous study, we found that women with MPI who had husbands living in cities other than their home village ceased having trance and possession experiences once they left their village to visit their spouse or father (23). However, if abuse in the family is a contributor to MPI outbreaks, it remains unclear why *chhopne* in children occurs almost exclusively in schools and not in the home [see (27)]. Perhaps *chhopne* episodes occur in school, not only because of a contagion effect, but also because it provides a safer setting for abused children to express their suffering and elicit help.

Disruptions in family dynamics associated with changes in family structure (i.e., from joint/extended to nuclear families) may also impede the development of secure attachment in children. Studies have found a strong association between attachment style and dissociative experiences in children, adolescents, and in later life [e.g., (75, 76)]. Further study of the links between changes in family configuration and developmental processes may help account for the apparent increase in MPI among adolescents in Nepal in recent decades.

### Current Distress

Social stress and psychological distress are commonly posited causes of MPI in the literature [e.g., (7, 17, 77)].This study did not find evidence for current distress as a predictor of case status. Although post-traumatic stress and depression were significantly associated with caseness in simple logistic regression, given the strength of association (i.e., Point Biserial Correlation (r_pb_) = 0.121, *p* < 0.05 and r_pb_ = 0.136, *p* < 0.05 for depression and PTSD, respectively), and the small amount of variance in case status accounted for by these variable (i.e., between 1.5% [Cox and Snell *R*^2^] and 2.5% [Nagelkerke *R*^2^]), it remains unclear when or to what extent trance and possession episodes experienced by adolescents in schools in Nepal can be viewed as a manifestation of underlying psychological distress or mental disorder. Further, the rates of distress in the non-MPI affected groups were high: 38.2% of non-affected children scored above the cut-off point (≥20) for post-traumatic stress and 27.4% scored above the cut-off point (≥14) for depression (45). This may reflect that fact that data for this study were collected 3–4 months after high-magnitude earthquakes in April and May 2015 in Nepal.

Although there are no studies assessing the comprehensive quality of life of individuals affected in MPI, factors indicating poor quality of life have been implicated in MPI outbreaks [e.g., (7, 13, 77, 78)]. However, quality of life was not a significant predictor of case status in the current study. It is likely that the selection of cases and controls with comparable demographics partially explains this result. As noted Van Ommeren et al. (20), it is possible that the affected children chose friends with similar backgrounds and situations as their control. The lack of association with quality of life also could be due to limitations of the instrument; we encountered several cross-cultural compatibility issues during the adaptation of this instrument [see (27)]. Given qualitative evidence in our earlier study, this finding should not be interpreted as demonstrating that stressors do not play a role in MPI, but rather suggests the need for more culturally and contextually sensitive measures of quality of life and social stress.

### Cognitive and Personality Trait Factors

Personality traits, except for hypnotizability, did not predict case status in MPI episodes. The associations between cognitive failures, fantasy proneness, emotional contagion and case status were not significant in bivariate as well as multivariable comparisons. To our surprise, the measure of cognitive failures was unrelated to MPI case status, although it was the strongest predictor of dissociative experiences and behaviors in the healthy adolescent population study (30) and was moderately correlated with dissociative behaviors and experiences (*r* = 0.39, *p*< *0.0*1) in this study (see Table 2). The results were also unexpected in terms of the role of emotional contagion in predicting caseness in MPI. Other researchers [e.g., (79, 80)] have hypothesized that individuals with higher susceptibility to emotional contagion are more likely to be affected in MPI outbreaks. The results of this study did not support this hypothesis. Emotional contagion was not significantly associated with caseness in MPI, either in bivariate or multivariable comparisons.

To our knowledge, no previous studies of MPI have used the measures of cognitive and personality traits employed in this study (i.e., cognitive failures, emotional contagion, fantasy proneness). Various studies have assessed other personality traits (e.g., neuroticism, extroversion, hypochondriasis, paranoia) among individuals affected in MPI. However, the findings have been inconclusive. Three reviews (2, 77, 78) concluded that case-control studies, in general, have not yielded useful results and that studies assessing personality factors in school outbreaks using the Minnesota Multiphasic Personality Inventory and Eysenck Personality Inventory were inconsistent. It is important to note, however, that cognitive failures, fantasy proneness, and emotional contagion were correlated with the measure of dissociative experiences and behaviors. This is consistent with the previous study on a general adolescent population in Nepal (30) as well as with studies conducted in the West [e.g., (32, 81)].

Hypnotizability emerged as the strongest predictor of MPI case status among the cognitive and personality trait variables. The results of this study are consistent with some of the previous studies [e.g., (82–84)] among college students and other adult population in the West. For example, hypnotizability was positively correlated with dissociative experiences and behaviors, emotional contagion (79), and post-traumatic stress [e.g., (85)] but not with childhood abuse [e.g., (86, 87)], depression, or cognitive failures. In contrast with the finding in the West that fantasy proneness and hypnotizability are associated (88, 89), there was no significant association between these factors in this study (see Table 2).

Hypnotizability and dissociation are distinct but related constructs (90). The literature on dissociation suggests that individuals who are highly hypnotizable are more likely to report dissociative experiences [e.g., (91)]. However, only a few previous studies have examined the hypnotic susceptibility or hypnotizability of children and adolescents affected in MPI (92, 93). Both, Lee et al. (92) and Tam et al. (93) reported a statistically non-significant difference in hypnotizability between affected and not affected children.

### Dissociative Experiences and Behaviors

Caseness in the MPI outbreaks was not associated with a general tendency to dissociate (as measured by the A-DES) but was with past experience of acute dissociation in response to a traumatic event. It is possible that individuals with higher trait dissociative tendency are more likely to experience peritraumatic (state) dissociation as a way of coping with psychological distress during abuse, and that those individuals who are already exposed to acute dissociation are more likely to be affected in MPI outbreaks in order to cope with the anxiety/fear produced by the perceived or real threat that triggered dissociative experience in the index case [e.g., see (94)]. Perhaps, as others have hypothesized [e.g., (32, 95)], among those who have experienced peritraumatic dissociation repeatedly “dissociation can presumably be automatized and invoked on a habitual basis in response to even minor stressors” [(32), p. 618; also see (96)].

### Limitations

This study has several limitations. First, the sample mainly included females, so gender-based comparisons were not possible. However, it should be noted that this was not because of any bias in sample selection and recruitment procedure, but because the number of affected male students was very low. Second, only three (e.g., DSRS, CPSS, BCTQ) of the instruments used had established validity and reliability in Nepal. Although we followed a rigorous translation and cultural adaption method and assessed test-retest reliability and internal consistency of the translated scales, which were acceptable for the majority of the instruments, there are no external validity measures for these instruments. Third, cases were selected based on reports of experiencing at least one *chhopne* episode during the MPI outbreak. No medical examination or psychological evaluation was conducted to identify or verify the cases. It is possible that some of the affected children were suffering from undiagnosed medical illness and that some children with *chhopne* episodes did not self-identify as such. Finally, although this study was conducted in five districts involving 12 MPI affected schools, considering the highly diverse ethnic and cultural composition of Nepal, the sample may not be representative of the whole country. A prospective study involving other ethnic and cultural groups, and assessing potential social-contextual factors, could address generalizability and might provide additional evidence for the social etiology of MPI outbreaks.

## Conclusion

In this case-control study of mass psychogenic illness in Nepal manifesting as trance and possession attacks among adolescents, the experience of possession (*chhopne*) had only a weak association with a measure of common dissociative experiences and behaviors among adolescents and with cognitive, personality and symptom measures that predicted other dissociative experiences and behaviors. This suggests that MPI may involve other processes in addition to those that account for individual experiences of dissociative symptoms or disorders.

An alternative approach to MPI, emerging largely from sociological research, suggests that the illness behaviors are culturally learned and patterned (97, 98) and may serve adaptive functions in the social contexts in which they occur. MPI has often been observed during times of social oppression, uncertainty, rapid cultural change, and political violence (17, 20, 99, 100). In the context of extreme oppression, MPI symptoms may function as a psychological defense or social expression of distress that is comprehensible in terms of local cultural models of affliction (101, 102). MPI symptoms may operate as local cultural idioms of distress, modes of communication that serve the afflicted individual's ongoing efforts to adapt to and survive in challenging social circumstances (103–106). Ethnographic studies have suggested, for example, that spirit possession may provide women or marginalized subgroups with the means to express distress in contexts where more direct expression is impossible or may have adverse effects (107, 108). Symptoms may allow an individual to draw attention to the personal conflict in a socially acceptable way (109–111), while the disavowal of causation and control serves to protect individuals from moral blame by positioning them as afflicted and in need of care.

The findings from this study have implications for our understanding potential causes and correlates of MPI. While some individual psychological variables appear to be important, key factors remain to be identified. In addition to exploring other psychological factors (e.g., secondary gain, suggestibility, absorption, expectancy, modeling, and behavioral mimicry), there is a need to examine social and cultural factors as well as school- and family-related factors (109). To identify potential factors, local perspectives on the nature, meaning and causes of *chhopne* also need to be elicited through qualitative and ethnographic studies. Future research should examine the role of contextual factors in the development of MPI outbreaks, including the rapid social, structural, and cultural changes taking place in Nepal today.

## Data Availability Statement

The datasets generated for this study are available on request to the corresponding author.

## Ethics Statement

The Research Ethics Committee of the Jewish General Hospital, Montreal Canada, provided ethical approval for this study. Considering low literacy of the parents/caregivers in rural settings of Nepal, we obtained verbal/oral consent of the parents or caregivers and with the agreement of the parents/caregivers, both the principal of the school, as a common guardian of the children during school hours, and the class teacher or an administrative staff, as a witness, provided written consent on the day of the interview. Students themselves provided informed assent.

## Author Contributions

RS and LK designed the study. RS took the responsibility for statistical analysis and drafted the manuscript. LK and AB supervised the analysis and substantially contributed to drafting and critical revision of the manuscript. All authors contributed to the article and approved the submitted version.

## Conflict of Interest

The authors declare that the research was conducted in the absence of any commercial or financial relationships that could be construed as a potential conflict of interest.

## Footnotes

^1^“Trance is a temporary alteration of consciousness, identity, and/or behavior evidenced by at least two of the following: (1) Marked alteration of consciousness or loss of the usual sense of identity without replacement by an alternate identity (2) Narrowing of awareness of immediate surroundings, or unusually narrow and selective focusing on environmental stimuli (3) Stereotyped behaviors or movements experienced as being beyond one's control” [Cardeña et al. (26), p. 173].
